# Theory of mind deficits in Parkinson’s disease are not modulated by dopaminergic medication

**DOI:** 10.3389/fneur.2023.1208638

**Published:** 2023-09-26

**Authors:** Tatiana Usnich, Elena Krasivskaya, Fabian Klostermann

**Affiliations:** Department of Neurology, Charité Universitätsmedizin Berlin, Berlin, Germany

**Keywords:** Parkinson’s disease, ToM, social cognition, levodopa, nonmotor symptoms

## Abstract

**Introduction:**

Patients with Parkinson’s disease (PD) exhibit deficits in social cognition, particularly with respect to Theory of Mind (ToM) capacities. It is unclear whether they are associated with PD-related dopamine deficiency and modulated by levodopa replacement therapy.

**Methods:**

A total of 15 persons with PD and 13 healthy controls (HC) participated in the study. They performed different neuropsychological tasks, including the Faux Pas Recognition Test (FPRT), assessing different dimensions of cognitive ToM (e.g., detection, inappropriateness, intentions), and the Reading the Mind in the Eyes Test (RMET) as an index of affective ToM. Persons with PD were tested twice, once under their regular treatment and another time after at least 18 h of levodopa withdrawal (MED-ON and MED-OFF, respectively). On either occasion, serum drug levels and motor symptom severity [Unified Parkinson’s Disease Rating Scale (UPDRS)] were measured.

**Results:**

MED-ON and MED-OFF conditions in patients with PD were confirmed by higher serum drug levels in the former than in the latter state and a corresponding amelioration of the motor deficit. In so doing, no performance difference in any ToM-related task was identified as a function of the levodopa therapy. Generally, patients performed worse than controls in both affective and cognitive ToM tests.

**Conclusion:**

Patients with PD have deficits in cognitive and affective ToM. Dopamine replacement, effective for improving the motor condition, does not appear to counteract these dysfunctions.

## Introduction

Parkinson’s disease (PD) is a neurological movement disorder. However, apart from typical motor symptoms such as bradykinesia, tremor, and rigidity, it implies numerous non-motor signs. Among the latter, deficits of social cognition (SC) are regularly associated with the disease. They could result from the spread of the neurodegenerative process beyond the typical loss of dopaminergic neurons within the nigrostriatal system, for example, to mesocortical networks.

SC refers to the cognitive operations necessary for socially adapted behaviors (1). A fundamental prerequisite of this capacity is the ability to generate own concepts of the mental states of other people (2), labeled as Theory of mind (ToM) (3). Commonly, ToM is divided into an affective part, underlying rather immediate, empathetic processes, and a cognitive aspect, comprising strategical inferences about given mindsets, e.g., as a model of intentions or motivations for perceived behaviors (4). Cognitive ToM strongly involves executive functions, such as updating, e.g., with respect to the concept of a social situation during its evolution, switching, e.g., between the perspectives of characters therein, and flexible informational retrieval, in order to construe complex behaviors as targeted action plans (5, 6). In contrast to this, affective ToM is considered an automatic process in which sensory input, e.g., from observed facial or bodily expressions, is related to mental representations of the own motor repertoire, conceivable as a process of imagery or reenactment (7, 8). Thus, the mechanisms by which PD compromises ToM could be different with respect to its distinct domains. In particular, a disorder of executive functions could contribute to cognitive ToM deficits, whereas the genuine motor deficit could also influence affective ToM performance if it extended to processes of internal motor simulation. Accordingly, the sensitivity of both aspects of ToM to PD treatment could be different.

In PD, deficits of cognitive and affective ToM have been described (9–11) and seem to grow together with disease progression (12). Several studies point to a particular involvement of dopamine deficiency in this regard. In animal studies, for example, amphetamine-induced dopamine release led to reduced affiliative social behavior, whereas antagonists of D1-type dopamine receptors reversed this effect (13). Furthermore, dopaminergic transmission in the mesocorticolimbic system, compromised in PD (14), modulates reward processing and, in so doing, is likely to exert effects on levels of social functioning (15). Concerning PD specifically, findings from patient studies on genuine disease effects and pharmacological replacement therapies appear heterogeneous. For example, ToM performance was described as both unaffected and abnormal in early PD, and particular effects of the pharmacological treatment were deemed absent (16, 17).

On the other hand, dopaminergic therapy was associated with the ability to interpret facial expressions correctly (18, 19). However, these and further investigations did not contrast the task performances of the same persons with PD in pharmacologically treated and untreated states. Hence, conclusions about real-life SC-related consequences of the disease and dopaminergic replacement therapy are difficult to draw. Accordingly, we tested the cognitive and affective components of ToM (20–22) in the same cohort of patients under their regular dopaminergic medication and after drug withdrawal. The results from these conditions were compared with each other and with the corresponding performances of persons without PD. We hypothesized that patients with PD perform worse than controls in cognitive and affective ToM tasks. Levodopa could, first of all, improve affective ToM deficits, given the mentioned results from animal research and a possible impact of motor system states on the decoding of bodily expressions including mimics. A secondary explorative aim was to further study cognitive task performances in either group and with respect to levodopa replacement in PD, given presumed associations of cognitive ToM with executive functions.

## Methods

### Study participants

Twenty-three patients with Parkinson’s disease (according to the criteria of the Movement Disorder Society) were enrolled in the study, treated only with levodopa with a decarboxylase inhibitor (Benserazide or Carbidopa) and recruited from the outpatient clinic for movement disorders at the Charité-Universitätsmedizin Berlin. The exclusion criteria were other known major psychiatric or neurological disorders and an unwillingness to undergo levodopa withdrawal. Eight participants did not complete the study protocol, of which six decided not to undergo the second test visit, one patient had a fall resulting in in-patient rehabilitation, and another one discontinued the medication and could, therefore, not be assessed under MED-ON. The data from 15 patients could finally be analyzed. From the pool of accompanying persons, 13 controls without Parkinson’s disease and free of any other neurological condition took part. All participants were over 18 years of age and had given written informed consent to the study protocol approved by the ethics committee of the Charité (EA4/165/17) in accordance with the Declaration of Helsinki.

### Cognitive screening

To assess the cognitive profile across different domains, patients under MED-ON and controls performed the Parkinson Neuropsychometric Dementia Assessment for cognition (PANDA-cognition). Similar to other screening tools, but designed for PD patients in particular, the PANDA-cognition comprises five subtests, i.e., (i) a word-learning task, (ii) alternating phonemic verbal fluency task, (iii) a visuospatial task, (iv) a working memory and attention task, and (v) delayed recall of the word list. Scores can range between 0 (worst) and 30 (best) points, with a cutoff for suspected dementia at values below 15. Additionally, the PANDA-mood was determined to assess the affective situation of the participants. The mood questionnaire consists of three questions assessing central aspects of depressive mood (mood, interest, drive) with a maximum score of 9 (23). Since, first of all, we aimed at examining potential intraindividual differences between ToM performances in the MED-ON versus MED-OFF condition within a realistic PD population, the PANDA was used to assess the cognitive profile of the participants without defining a cut-off value for study exclusion. With respect to controls, we sought to reach an acceptable match of the PANDA values between the groups.

Patients were tested twice at intervals of at least 2 months, on the one hand, under their regular levodopa treatment (MED-ON), and on the other hand, at least 18 h after their last levodopa intake (MED-OFF). The order of the examination days per condition (first MED-ON, second MED-OFF/first MED-OFF, second MED-ON) was balanced between the patients. Patients ran two task versions for further cognitive tests, one under MED-ON and the other under MED-OFF, starting with version 1 or 2 in random order.

### Assessment of affective and cognitive theory of mind

The participants engaged in two standard theory of mind (ToM) tasks: the Reading the Mind in the Eyes Task (RMET) and the Faux Pas Recognition Test (FPRT). In the RMET, one has to decide about emotional states based on photos of the periocular eye region of different persons (one photo per person). The decision is made from four predefined options (e.g., angry, sad, friendly, and flirty). Since the task requires the perceptual decoding of facial expressions, the RMET measures empathetic capacities, i.e., affective ToM (24).

For the patients, the original set of 36 pictures was divided into two parts of 18 photos in order to not repeat presentations in either MED-ON or MED-OFF but to have them engage in the entire task over the two sessions. Controls (having one test session only) ran the original task. Scores were expressed as the percentage of correctly evaluated pictures from all the presented pictures. Unlike the RMET, the FPRT is text-based. Although the last question in the FPRT task addresses an aspect of affective ToM, the entire task paradigm demands cognitive ToM capacity, implying strategic thinking and perspective changes (16, 20). The task comprises 20 short stories in which persons communicate with each other. Half of them contain conversations with inappropriateness regarding social rules, and the other half do not. Per story, one has to answer (i) whether the misconduct was present (Faux Pas Detection), and, if this was the case, (ii) why it is inappropriate (Faux Pas Inappropriateness), (iii) which goal it pursued (Faux Pas Intention), (iv) whether it was formulated accidentally or on purpose (Faux Pas Belief), and (v) which emotions it triggered in the interlocutors (Empathy). For correct (incorrect) responses, one (no) point is given; scores are expressed as the percentage ratio of reached to maximally possible points. The stories in the FPRT task were presented to the patients on paper and read aloud by the examiner. The answers were given verbally and noted down by the examiner. There was no time limit for the responses to the questions since the task focus was on accuracy. PD patients engaged in split test versions with 10 stories per MED-ON and MED-OFF, respectively (containing 5 stories with and without a faux pas each), so they ran the entire task over the two test sessions. Controls performed the original task in one session.

### Further cognition assessment

The PD patients performed several additional tasks to assess whether potential changes in ToM performance by levodopa intake were associated with modulations of other cognitive functions. Specifically, the Cognitive Subscale of the Alzheimer’s Disease Assessment Scale (ADAScog) was used, comprising subtasks for word recall and recognition, naming objects and fingers, following commands, constructional and ideational praxis, orientation, language capacities, and memory (25) Furthermore, a number of dedicated functions of interest, typically compromised in PD, were tested with the digit span forward and backward tests (26), the clock drawing test (27), the German standard test for verbal fluency (Regensburger Wortflüssigkeitstest, RWT) (28), and the concept shifting test (CST) (29). The digit span forward and backward tests were performed to assess working memory and attention. The examiner verbally presented a span of three to nine digits and captured the participants’ verbal recall. Testing was stopped after two consecutive failures of the same span length. In the RWT, participants were asked to name as many words as possible starting with “S” (set 1) or “K” (set 2; phonematic verbal fluency) or to name animals (set 1) or groceries (set 2; semantic verbal fluency) in 1 min. The naming of erroneous words was not counted. For the CST, paper sheets with 16 small circles arranged in a larger circle were presented. The small circles contained digits, letters, both digits and letters, or were empty. Participants were instructed to cross out as quickly as possible the randomly arranged numbers in numerical order, the letters in alphabetical order, and the numbers and letters alternatingly in numerical and alphabetical order, respectively (e.g., 1 A 2 B 3 C).

Alternative versions were run for all tasks in the MED-ON and MED-OFF conditions.

### Assessment of the motor condition and drug levels in patients

To determine the patients’ movement condition as a function of levodopa intake, the motor part of the Unified Parkinson Disease Rating Scale (UPDRS Part III) in the MED-ON and MED-OFF conditions was used. UPDRS scores range from 0 to 108, with lower scores representing less severe motor impairment. Furthermore, serum levels of levodopa and oximethyl-DOPA (metabolite) were assessed in blood samples taken in either condition. As the plasma half-life of levodopa is up to 2 h, the levodopa levels were expected to be hardly detectable under MED-OFF. However, as the effects of levodopa have been described for up to 2 weeks after levodopa withdrawal, we analyzed oximethyl-DOPA, a major metabolite of levodopa, possibly indicating prolonged levodopa effects.

### Data analysis

Normal data distribution was analyzed by the Kolmogorov–Smirnov and Shapiro–Wilk tests with further assessment of skewness and kurtosis. For normally distributed variables, between-group and within-group comparisons were performed by corresponding *t*-tests; otherwise, non-parametric tests for independent samples (Mann–Whitney *U*-Test) or for related samples (Wilcoxon Signed-Rank Test) were used. To determine whether there was evidence for the absence of the effect, we conducted Bayesian statistical analyses. The significance level was set at *p*-values < 0.05. For data analysis, the software package SPSS, Version 27.0.0.0, was used (IBM Corp., Armonk, NY, United States).

## Results

### Clinical and demographic characteristics

PD patients and controls did not significantly differ with respect to age, sex, and duration of school education. PANDA scores did not statistically differ between the groups. Still, the group contrast of PANDA-cog values almost reached significance. PD patients showed lower scores than healthy controls in line with known, foremost executive deficits compared to the age-matched persons. PD patients were treated by levodopa with a dopamine decarboxylase inhibitor (Benserazide or Carbidopa). The mean interval between the test sessions in the MED-ON and MED-OFF conditions was 72 days (± 66 days). Clinical and demographic characteristics are displayed in Table 1.

**Table 1:** Clinical and demographic characteristics of study participants.

	PD patients (*N* = 15)	HC (*N* = 13)	95% CI	*t*(df)	*p*
Age at enrolment, mean (± SD)	75.33 (± 8.98)	73.85 (±5.74)	−4.478-7.453	0.512 (26)	0.613
Sex (m), *n* (%)	10 (66.7%)	7 (53.8%)	-	0.480 (1)	0.700
Years of education, mean (± SD)	11.00 (± 2.54)	10.15 (± 2.15)	−0.998-2.690	0.943 (26)	0.354
PANDA Cog, mean (± SD)	20.67 (± 5.97)	25.00 (± 5.08)	−8.679-0.012	−2.050 (26)	0.051
PANDA Mood, mean (± SD)	2.86 (± 2.32)	1.69 (±1.75)	−0.472-2.802	1.465 (25)	0.155
Disease duration, y, mean (± SD)	4.13 (± 3.83)	**-**			
H&Y, median (range)	2.0 (1.0–4.0)	**-**			

PD, Patients with Parkinson’s disease; HC, healthy controls; *p*, value of *p*; SD, standard deviation; *t*, test statistics; df, degrees of freedom; 95% CI, confidence interval; m, male; n, number; PANDA, Parkinson Neuropsychometric Dementia Assessment; MADRS, Montgomery–Åsberg Depression Rating Scale; H&Y, Hoehn and Yahr.

### Patients’ motor condition and drug levels in MED-ON and MED-OFF conditions

Levodopa [*t*(df) = 3.703 (13); *p* = 0.003] and oximethyl-DOPA [*t*(df) = 4.980 (14); *p* < 0.001] levels differed significantly between the MED-ON and MED-OFF conditions of the patients. Whereas levodopa was almost absent in the MED-OFF condition, oximethyl-DOPA was still detectable in the MED-OFF condition but significantly lower than in the MED-ON condition. Accordingly, the motor condition was significantly worse in the MED-OFF than in the MED-ON condition, as indicated by higher scores in the UPDRS Part III [*t*(df) = −6.022 (14); *p* < 0.001; see Table 2].

**Table 2:** Levodopa levels and motor examination in PD patients under MED-ON and MED-OFF.

	PD MED-ON	PD MED-OFF	95% CI	*t*(df)	*p*
Levodopa in mg/L, mean (±SD)	0.464 (±0.4684)	0.007 (±0.0267)	0.1904–0.7239	3.703 (13)	**0.003**
Oximethyl-DOPA in mg/L, mean (±SD)	4.933 (±2.8389)	2.000 (±2.1173)	1.6701–4.1966	4.980 (14)	**<0.001**
UPDRS III, mean (±SD)	22.40 (±5.539)	32.27 (±9.505)	−13.381—6.353	−6.022(14)	**<0.001**

PD, Patients with Parkinson’s disease; *p*, value of *p*; SD, standard deviation; *t*, test statistics; df, degrees of freedom; 95% CI, confidence interval; UPDRS III, Unified Parkinson’s Disease Rating Scale Part III. Bold values indicate significant test results.

### Performance on theory of mind tests

**Figure 1:**
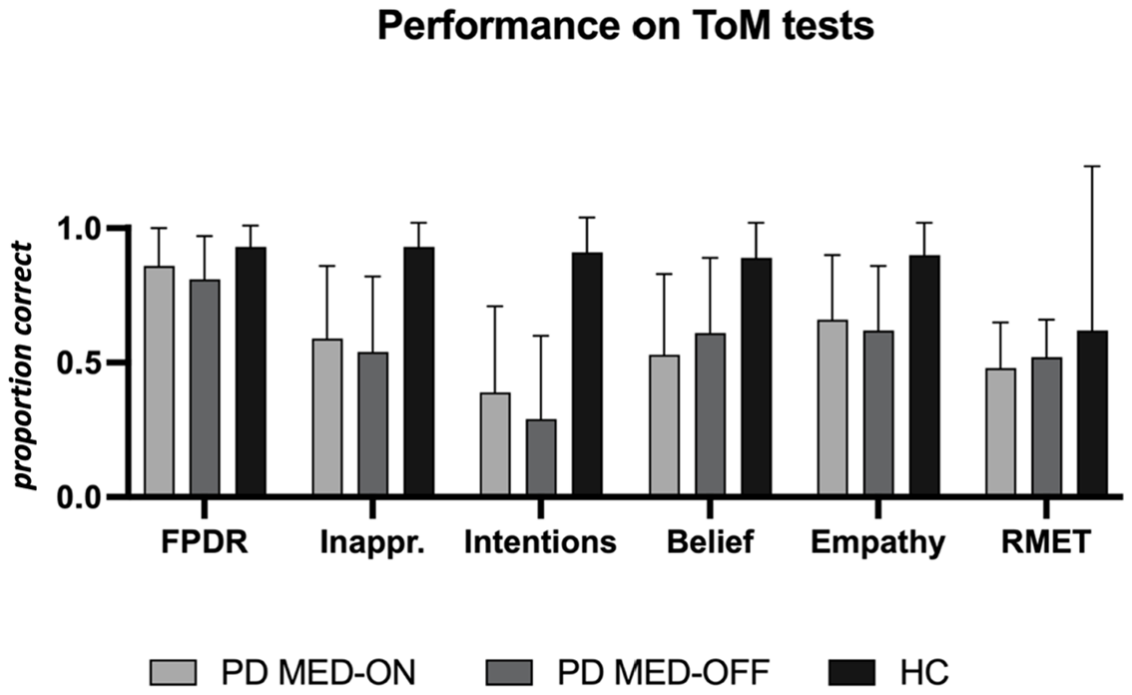
Performance on theory of mind tests. PD MED-ON, Patients with Parkinson’s disease on levodopa; PD MED-OFF, Patients with Parkinson’s disease off levodopa; HC, healthy controls; FPDR, Faux pas discovery rate; Inappr., Understanding inappropriateness; RMET, Reading the mind in the eyes test.

The results of the FPRT were not normally distributed. The faux pas detection rate was lower in patients under MED-OFF than in controls (T = 146; r = 0.082; *p* = 0.025). This statistical group difference vanished when patients were in the MED-ON condition. Concerning all other cognitive theory of mind dimensions (understanding inappropriateness, intentions, belief, and empathy), patients, be they under MED-ON or MED-OFF, performed worse than controls. Within patients, no performance differences were identified between the MED-ON and MED-OFF conditions (Figures 1 2).

**Figure 2:**
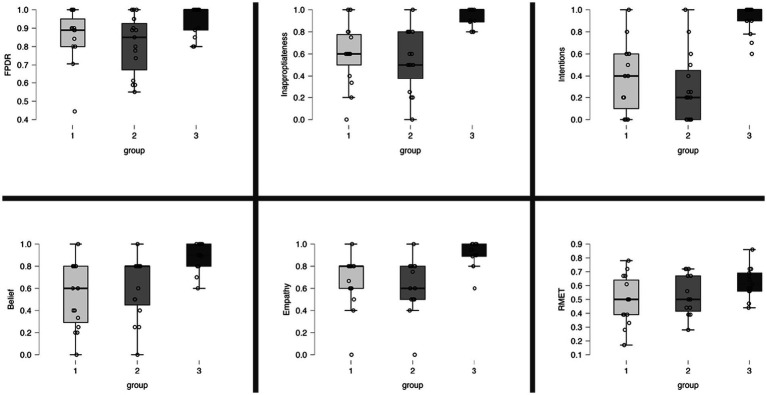
Overview of individual results per ToM task. Proportion of correct responses in the ToM tests. Boxes represent interquartile range and the median. Group 1: PD, MED-ON; Group 2: PD, MED-OFF; Group 3, Healthy controls; FPDR, faux pas detection rate; RMET, Reading the mind in the eyes test.

The RMET results were lower in the patients under MED-ON than in controls [*t*(df) = 2.453 (26); *p* = 0.021]. However, under MED-OFF, this group difference failed to be significant, with the difference between MED-ON and MED-OFF performances being marginal (Figures 1 2; Table 3).

**Table 3:** Theory of mind test results in patients and controls.

	PD MED-ON	PD MED-OFF	HC	PD MED-ON vs. HC, test statistics	PD MED-ON vs. HC, *p*	PD MED-OFF vs. HC, test statistics	PD MED-OFF vs. HC, *p*	PD MED-ON vs. MED-OFF, test statistics	PD MED-ON vs. MED-OFF, *p*
FPDR, mean (±SD)	0.86 (±0.14)	0.81 (±0.16)	0.93 (±0.08)	T = 131	0.130	T = 146	**0.025**	T = 38.5	0.379
				r = 0.057		r = 0.082		r = −0.029	
Understanding inappropriateness, mean (±SD)	0.59 (±0.27)	0.54 (±0.28)	0.93 (±0.09)	T = 173	**<0.001**	T = 179.5	**<0.001**	T = 22	0.574
				r = 0.127		r = 0.138		r = −0.019	
Intentions, mean (±SD)	0.39 (±0.32)	0.29 (±0.31)	0.91 (±0.13)	T = 180.5	**<0.001**	T = 182	**<0.001**	T = 14.5	0.182
				r = 0.139		r = 0.141		r = −0.045	
Belief, mean (±SD)	0.53 (±0.30)	0.61 (±0.28)	0.89 (±0.13)	T = 169	**<0.001**	T = 163.5	**0.002**	T = 15	0.386
				r = 0.119		r = 0.111		r = 0.029	
Empathy, mean (±SD)	0.66 (±0.24)	0.62 (±0.24)	0.90 (±0.12)	T = 168	**<0.001**	T = 172.5	**<0.001**	T = 26	0.532
				r = 0.119		r = 0.125		r = 0.021	
RMET, percentage, mean (±SD)	0.48 (±0.17)	0.52 (±0.14)	0.62 (±0.61)	*t*(df) = −2.453 (26)	**0.021**	*t*(df) = −2.050 (26)	0.051	*t*(df) = −0.714 (14)	0.487
				95% CI = −0.255–0.224		95% CI = −0.204-0.000		95% CI = −0.148–0.743	

PD, Patients with Parkinson’s disease; HC, healthy controls; *p*, value of *p*; SD, standard deviation; *T*, test statistics; df, degrees of freedom; 95% CI, confidence interval; r, effect size; FPDR, faux pas detection rate; RMET, Reading the Mind in the Eyes Test. Bold values indicate significant test results.

In Bayesian statistics, the likelihood of performance differences between MED-ON and MED-OFF being absent was three to four times higher than the likelihood of these differences being present [FPDR (BF_01_ = 3.209; *p* = 0.343), Understanding inappropriateness (BF_01_ = 4.018; *p* = 0.481), Intentions (BF_01_ = 3.099, *p* = 0.313), Belief (BF_01_ = 3.786; *p* = 0.433), Empathy (BF_01_ = 4.331; *p* = 0.557), RMET (BF_01_ = 4.046, *p* = 0.487)].

### Neuropsychological assessment

The results from the further neuropsychological assessments (ADAScog, the digit span forward and backward, the clock drawing test, semantic and phonematic verbal fluency tests, and concept shifting tests) were not significantly different between the MED-ON and MED-OFF conditions in PD patients (Table 4).

**Table 4:** Neuropsychological tests MED-ON and MED-OFF.

	PD MED-ON	PD MED-OFF	95% CI	*t*(14)	*p*
ADAS memory, mean (±SD)	6.93 (±3.369)	6.87 (±3.871)	−1.265–1.398	0.107	0.916
ADAS cognition ex memory, mean (±SD)	2.60 (±2.230)	2.87 (±2.031)	−0.944–0.410	–0.845	0.413
ADAS sum cog, mean (±SD)	9.53 (±5.276)	9.73 (±4.949)	−1.698–1.298	–0.286	0.779
ADAS sum noncog, mean (±SD)	5.60 (±5.040)	5.40 (±4.205)	−1.383–1.783	0.271	0.790
ADASUM, mean (±SD)	15.13 (±6.534)	15.13 (±5.069)	−2.546–2.546	0.000	1.000
Digit span forward, mean (±SD)	6.87 (±2.100)	6.80 (±2.305)	−0.808–0.941	0.163	0.872
Digit span backward, mean (±SD)	4.73 (±2.154)	5.40 (±1.682)	−1.495–0.162	–1.726	0.106
Clock, mean (±SD)	1.87 (±0.990)	2.20 (±1.265)	−0.785–0.119	–1.581	0.136
Verbal fluency, sem, mean (±SD)	19.40 (±6.434)	17.60 (±4.579)	−1.647–5.247	1.120	0.282
Verbal fluency, phonem, mean (±SD)	12.33 (±5.287)	11.53 (±4.224)	−1.367–2.967	0.792	0.442
CST* num, mean (±SD)	21.73 (±10.19)	17.30 (±5.97)	−0.115–8.878	2.139	0.051
CST* let, mean (±SD)	29.60 (±21.13)	31.23 (±13.77)	−11.588–8.321	–0.352	0.730
CST* switch, mean (±SD)	45.33 (±30.95)	60.30 (±65.88)	−37.317–7.384	–1.436	0.173
CST* shifting score, mean (±SD)	19.67 (±21.01)	36.03 (±63.77)	−43.503–10.769	−1.294	0.217

PD, Patients with Parkinson’s disease; *p*, value of *p*; SD, standard deviation; *t*, *t*-test value; CI 95%, confidence interval; ADAS, Alzheimer’s Disease Assessment Scale; CST, Concept Shifting Test; *, time needed for accomplishing the task.

## Discussion

Persons with PD performed worse than controls in cognitive and affective ToM tasks, in line with previous reports on social cognitive deficits in this condition. Dopamine replacement therapy did not exert a relevant impact on these dysfunctions.

Whether a therapy partially compensating for the central neurotransmitter deficit in PD and robustly reducing motor symptoms also improves relevant non-motor dysfunctions is an obvious question, which, with respect to ToM, has not been studied in the same patients in the MED-ON and MED-OFF conditions. Absent levodopa effects on ToM dysfunctions in PD fall in line with the observation that cognitive changes of PD are largely unresponsive to dopaminergic treatment (30). This is worthwhile to note since ToM may, at least partly, reflect processes relatively independent of other cognitive functions (31), but also because different data and concepts suggest its susceptibility to PD medication. For example, dopamine-releasing drugs weakened affiliative behaviors in animal studies, and this effect vanished after pharmacological dopamine receptor blockade (13). Furthermore, in PD, dopamine transmission is not only deficient in the nigrostriatal system, associated primarily with motor function, but also in mesocorticolimbic networks (14), presumably involved in processing social cognitive functions (15, 32). Previously, subtle differences in the recognition of negative facial expressions were reported with respect to unmedicated versus medicated PD patients (18, 19), whereby it has to be noted that these groups differed largely with respect to disease parameters (e.g., the medicated group was in a more advanced disease stage). A theoretical basis for the assumption of levodopa-related effects on ToM dysfunctions in PD comes from cognitive embodiment concepts. In this view, internal simulation or imagery is conceived to be instrumental in the semantic decoding of, e.g., observed gestures, postures, or facial expressions, providing a quasi-experience of the perceived (33, 34). In this vein, a human mirror neuron network implying primary motor regions was proposed based on brain activations during mere movement observation (35–37). From this perspective, levodopa-induced improvement of ToM via enhancing motor system state functions in PD seems conceivable.

However, the current data suggest that levodopa has no relevant impact on ToM dysfunctions under realistic therapeutic conditions. This analysis is supported by the Bayesian statistics finding the likelihood of absent differences in the MED-ON versus MED-OFF conditions three to four times higher than the likelihood of their presence. Nevertheless, considering several study limitations, minor effects cannot be ruled out. Due to the fact that affected persons are mostly under different PD drugs, candidates on a monotherapy able to undergo the test protocol were altogether rare, so the group size remained small with 15 patients. Given that in trials with comparable research questions similar numbers were reported (16–19), further studies on related topics might therefore consider multicenter recruitment strategies from the beginning to overcome this shortcoming. Furthermore, levodopa was reported to unfold verifiable motor effects even weeks after drug withdrawal, suggesting that its serum levels do not necessarily represent central drug actions (38). Therefore, we additionally assessed oximethyl-dopa as an active metabolite, decreased in the MED-OFF compared to the MED-ON condition, but naturally, it is theoretically possible that levodopa effects on ToM could become evident only after longer drug withdrawal phases. However, longer intake pauses would not have been feasible since one-third of the recruited patients dropped out for excessive strain caused by the demanded drug withdrawal.

Since patients in the PD group underwent two test sessions, they only performed one-half of the ToM task trials per measurement, so they altogether went through the same tasks as the controls did in one session. Thus, the shorter test series in the PD patients than in the controls could theoretically have influenced the results, e.g., in that learning occurs throughout task performance. However, no difference between the ToM performances in the first compared to the second session of ToM task performances was identified in the PD patients. Therefore, we think the disparity of ToM testing per session did not relevantly influence the current results. However, the exact behavioral meaning of the FPRT and RMET results remains to be settled. For example, cognitive ToM functions, such as first- and second-order beliefs, are not differentiated by the FPRT, and the narration of social situations or the presentation of static pictures differs from dynamic and polymodal event perception in real life. In this regard, the development of dedicated task designs and the comparison of test outcomes with indices of natural social conduct are a need that should be addressed in future studies.

Finally, concerning potential subtle effects, it deserves mention that, compared to the controls, the patients’ performance in the RMET was only statistically abnormal under MED-ON and in one dimension of the FPRT only under MED-OFF (the faux pas detection rate, FPDR). This is theoretically compatible with task-specific levodopa effects. According to the overdose hypothesis, levodopa replacement compensates for the overall dopamine deficit in dorsal striatal networks but may flood better-preserved ventral striatal regions (39). The latter form part of networks implying the ventromedial prefrontal and orbitofrontal regions, which are involved in affective ToM processing (40, 41), whereas the dorsal striatum projects to dorsolateral prefrontal areas with a role in cognitive ToM (10, 21). From this perspective, levodopa-induced worsening of RMET performance seems conclusive, just as a (cognitive ToM-related) FPDR improvement. However, given their almost negligible size, corresponding intraindividual differences need to be corroborated in further studies and are, for the time being, not deemed clinically relevant.

We conclude that levodopa replacement does not unfold relevant effects on ToM dysfunctions in PD. This inertness of social cognitive deficits to the pharmacological mainstay of PD treatment is unfortunate but is in parallel with the behavior of further cognitive dysfunctions characteristic of the condition.

## Data availability statement

The raw data supporting the conclusions of this article will be made available by the authors, without undue reservation.

## Ethics statement

The studies involving humans were approved by Ethics Committee of the Charité Universitätsmedizin Berlin. The studies were conducted in accordance with the local legislation and institutional requirements. The participants provided their written informed consent to participate in this study.

## Author contributions

TU: research project: conception, organization, and execution, statistical analysis: design and execution, and manuscript: writing of the first draft. EK: research project: organization and execution, statistical analysis: review and critique, and manuscript: review and critique. FK: research project: conception, organization, and execution, statistical analysis: design and review and critique, manuscript: writing and review and critique. All authors contributed to the article and approved the submitted version.
