# Erratum: Theory of mind deficits in Parkinson's disease are not modulated by dopaminergic medication

**DOI:** 10.3389/fneur.2023.1351404

**Published:** 2023-12-19

**Authors:** Frontiers Production Office

**Affiliations:** Frontiers Media SA, Lausanne, Switzerland

**Keywords:** Parkinson's disease, ToM, social cognition, levodopa, nonmotor symptoms

Due to a production error, the funder “Jiangsu Agri-Animal Husbandry Vocational College” was erroneously inserted.

The publisher apologizes for this mistake. The original article has been updated.

